# Rapid implementation of home oxygen treatment and remote monitoring for COVID-19 patients at the verge of the Omicron wave in Turku, Finland

**DOI:** 10.1186/s12879-023-08825-5

**Published:** 2023-11-15

**Authors:** Janne Hänninen, Ulla Anttalainen, Maritta Kilpeläinen, Ulla Hohenthal, Niklas Broman, Jenni Palmén, Jarmo Oksi, Thijs Feuth

**Affiliations:** 1 Department of Pulmonary Diseases and Allergology, Division of Medicine, University of Turku and Turku University Hospital, Turku, Finland; 2 Department of Infectious Diseases, University of Turku and Turku University Hospital, Turku, Finland; 3 Turku City Hospital, Turku, Finland

**Keywords:** COVID-19, SARS-CoV-2, Telemedicine, Hypoxemia, Remote oxygen treatment, Omicron

## Abstract

**Background:**

In Turku, Finland, we introduced a home oxygen treatment and app-based monitoring program for hospitalized COVID-19 patients to facilitate an early discharge during the Omicron wave. In this case series we explore the clinical parameters of patients enrolled in the program and evaluate the cost–benefit and safety issues of the program.

**Methods:**

Hospitalized COVID-19 patients with marked hypoxemia but otherwise in stable condition were screened from Turku City Hospital and Turku University Hospital by treating doctors for eligibility in the program. Peripheral oxygen saturation of > 92% and breathing frequency < 30/min in rest with oxygen supplementation were among the criteria. All patients actively participating in the program between 10^th^ of January 2022 and 30^th^ of September 2022 were included in this case series. Clinical data of hospitalization and monitoring were analysed, and cost–benefit evaluation was based on the number of saved hospitalization days.

**Results:**

Nineteen COVID-19 patients were included in this case series and recruited from three different hospital departments in the Turku city region, South-West Finland. All patients were male, the median age was 59 years and the median duration of hospitalization before enrolment in the program was 6 days (range 3—20 days). The median duration of home oxygen treatment was 13 days (range 3—72 days) and the median duration of home monitoring was 18 days (range 7—41 days). A total of 210,5 hospital days were prevented, resulting in savings of €144,490 of healthcare expenditure (on average 9 days and €7,605 per patient). No major safety issues were reported during the program.

**Conclusions:**

In our case series, home oxygen treatment combined with home monitoring was safe and economically beneficial. Application based monitoring could be considered in other post-acute pulmonary conditions to reduce hospitalization and healthcare costs.

## Background

The COVID-19 pandemic caused by severe acute respiratory syndrome coronavirus 2 (SARS-CoV-2) has overwhelmed healthcare systems all over the world [1]. In Finland, the timely deployment of preventive strategies has limited the burden of COVID-19 and Finland proved to be one of the most resilient European countries during the first two years of the pandemic [2]. By the end of 2021, 1510 people of a total population of approximately 5.6 million had died due to COVID-19 (i.e. 0.03% of the population) [3]. By the 12^th^ of January 2022, 81.5% of the population over five years of age had received at least one dose of a highly effective vaccine against COVID-19 [4]. However, hospital admissions accelerated during the first months of 2022 due to the introduction of the Omicron variant, despite the decreased risk of severe COVID-19 on an individual level in comparison with the previously dominant Delta variant [5]. The effective reproductive number of Omicron variants was several times the previous Delta variant and vaccination induced prevention against symptomatic infections was reduced due to viral mutations [5].

A substantial proportion of hospitalized COVID-19 patients require prolonged oxygen treatment. In several countries, telemedicine has been deployed to enable home follow-up with or without oxygen treatment and to facilitate early discharge form hospital care during the early phase of the pandemic [6–10]. To our knowledge, no such program has been initiated in Finland before 2022. As we anticipated accelerating numbers of hospital admissions at the verge of the Omicron wave, we rapidly implemented a home oxygen delivery service for COVID-19 patients in a collaboration project between Turku City Hospital and Turku University Hospital in South-West of Finland. In this study, we investigate the utility of the program.

## Patients and methods

### Study design

In this two-center case series, we describe the introduction of a home monitoring and oxygen delivery program deploying telemedicine for COVID-19 patients as a pandemic relieve measure, and retrospectively analyze treatment results and patient safety issues of patients enrolled in the program. Furthermore, we perform a cost–benefit assessment of the remote home oxygen treatment and monitoring program.

### Patients and study sites

For enrollment, patients were selected from COVID-19 department of Turku City Hospital and from Pulmonary Diseases- and Infectious Diseases-Departments of Turku University Hospital. All patients enrolled in the program between 10^th^ of January 2022 and 30^th^ of September 2022 were included in this study.

### Eligibility of patients for enrollment in the program

All patients hospitalized for COVID-19 were screened for eligibility for the program, guided by inclusion and exclusion criteria listed in Table 1. Screening was performed by the treating physicians at the study sites.

**Table 1:** Inclusion- and exclusion criteria for participation in the home oxygen treatment and monitoring program

Inclusion criteria	Exclusion criteria
- Recovering from severe COVID-19 - SpO2 > 92% and breathing frequency < 30/min in rest with oxygen supplementation - No immediate risk of death without oxygen supplementation - Safety measures for home oxygen treatment fulfilled - No severe problems with the treatment or monitoring expected at home	- Progressive COVID-19 - Clinically unstable - High flow oxygen support - Fire hazards at home - Multiple severe underlying conditions - Inability to take food and drugs orally - Inadequate cooperation or safety not guaranteed

### Monitoring during home treatment

Hospitalized patients were monitored with a mobile phone application developed by a commercial partner HealthFOX. Before hospital discharge, the patients received guidance to the use of the mobile application, an oxygen enrichment device, a pulse oximeter, and blood pressure monitor from healthcare workers. Symptoms were assessed daily by a questionnaire and vital measurements were reported three times a day via the mobile application. Reported data was checked thrice daily by clinical staff of the Pulmonary Diseases Department of Turku University Hospital. The application included a chat function, which was answered in real time during office hours and checked regularly at set times outside working hours, as well as a video call function. Patients were provided with a phone number for acute situations within and beyond working hours and were instructed to call the emergency number 112 in case of possible emergencies. Assessment of rapid availability of help in case of emergencies was part of the overall safety assessment. For safety, the application included cut-off limits for the vital measurement values in addition to the possibility to chatting or calling and the daily checking by the treating staff. Basic laboratory evaluation of inflammation markers was performed on the sixth day of home oxygen treatment.

### Study outcomes

For this retrospective analysis, baseline clinical parameters, including patient demographics and the severity of COVID-19, as well as outcome data such as oxygen treatment and duration of monitoring were explored.

For the cost–benefit analysis, two pulmonologists (UA and TF) with expertise in COVID-19 hospital care estimated independently, based on clinical parameters, the probable date of discharge from the hospital had home oxygen treatment not been initiated. Three cases with marked difference (5 days or more) between the two evaluations were discussed among both pulmonologists and then re-evaluated until agreement was achieved. The mean of the two estimations was used as the theoretical day of discharge to calculate the avoided hospitalization expenditure. According to hospital guidelines, isolation was applied during the first 10 days of disease, beginning from the onset of symptoms. In accordance with the costs of care listing for the patients’ home municipalities, the costs of hospitalization were €910 per day in isolation and €850 per day not in isolation care in Turku University Hospital.

### Statistical analysis

Descriptive analysis was used for demographics and patients’ clinical characteristics. These were expressed either as frequencies and percentages or median with interquartile range (IQR) as not all the data was distributed normally. Statistical analysis was performed with SPSS, version 28 (IBM SPSS).

## Results

### Design and initiation of the program

Despite high uptake of COVID-19 vaccination among the population of Finland, high impact of the newly discovered Omicron was anticipated on by the end of 2021. A first meeting to evaluate feasibility of an oxygen treatment and monitoring program was held on the 16^th^ of December 2021 with COVID-19 treating specialists of both study centers and representatives of regional and ambulant health services. Development of the protocol and an online application was immediately initiated, with active involvement of nurses and medical doctors treating COVID-19 patients in order to utilize their professional skills and to promote a positive attitude towards the rapid introduction of the new intervention. In addition to assessment of available literature, international networks were employed to learn from experiences in several other countries and we received protocols of similar programs which had been earlier initiated elsewhere. After education of healthcare professionals, the program was ready to be launched at 10^th^ of January 2022.

### Study population

**Fig. 1:**
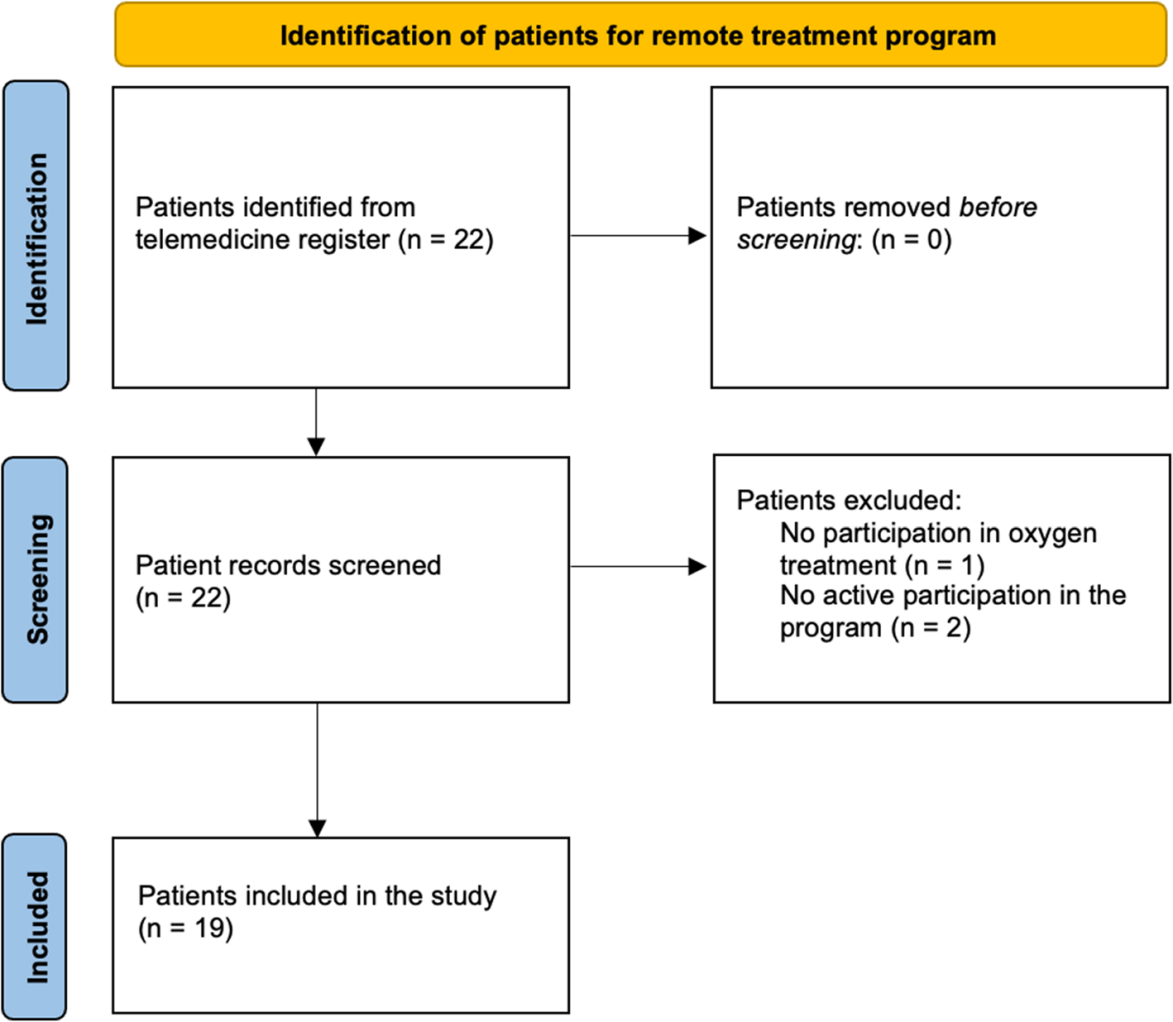
Flow chart for patient inclusion

From the beginning of January 2022 to the end of September 2022, 22 COVID-19 patients were recruited for participation in the home oxygen treatment program after screening by treating physicians, as depicted in Fig. 1. Of all, one patient participated in the follow-up without oxygen treatment because of a high C-reactive protein (CRP) value without any need of supplemental oxygen, and two patients took part in the follow-up and home oxygen treatment but did not actively participate in the program and were thus excluded. Therefore, the total study population consisted of nineteen patients of whom all were male with a median age of 59 years. The median symptomatic period before hospital admission was 10 days ranging from 1 to 14 days. Two patients (12%) were current smokers, nine (53%) were ex-smokers and 10 patients (59%) had received at least one vaccine dose against COVID-19. Five patients (26%) did not have any of the diseases specified in Table 2. Eighteen patients (95%) had some underlying medication.

**Table 2:** Baseline characteristics of patients included in the study

	Median value or n/N	Q1-Q3 or %
Male gender	19/19	100%
Age, years	59	51 – 68
Smoking		
Current smoker	2/17	12%
Ex-smoker	9/17	53%
Non-smoker	6/17	35%
Body mass index (BMI), kg/m^2^	29.3	26.6 – 31.2
SARS-CoV-2 vaccination		
Unvaccinated	7/17	41%
At least 1 vaccine dose	10/17	59%
Comorbidities		
No comorbidities	1/19	5%
Hypertension	11/19	58%
Obesity (BMI ≥ 30)	7/18	39%
Diabetes mellitus	7/19	37%
Chronic kidney failure	5/19	26%
Haematologic disease	2/19	11%
Asthma or COPD	2/19	11%
Rheumatic disease	1/19	5%
Sleep apnea	1/19	5%
Paroxysmal atrial fibrillation	1/19	5%
Other, unclassified	5/19	26%
Pre-existing DNR	2/19	11%
Long-term medication before admission		
Oral glucocorticoid	3/19	16%
Inhaled glucocorticoid	2/19	11%
Anti-rheumatic medication	2/19	11%
Anticoagulants	3/19	16%
Other medicines	18/19	95%
Findings upon admission		
Duration of symptoms before admission, days	10	3 – 14
Peripheral oxygen saturation, %	91%	87% – 95%
Heart rate, beats/minute	100	85 – 108
Systolic blood pressure, mmHg	136	103 – 149
Diastolic blood pressure, mmHg	74	59 – 84
Leukocytes, × 10^9^/L	6.6	5.3 – 9.2
Neutrophils, × 10^9^/L	5.3	4.5 – 6.8
Lymphocytes, × 10^9^/L	1.0	0.6 – 1.4
C-reactive protein, mg/L	82	57 – 151
Ferritin, µg/L	1158	560 – 1793
Procalcitonine, µg/L	0.21	0.12 – 0.36
D-dimer, mg/L	0.40	0.30 – 0.80
Interleukin-6, pg/ml	33	17–81
Myxovirus resistance protein A > 800 µg/L	12/17	71%
Detectable SARS-CoV-2 specific IgG	14/19	74%

*COPD* chronic obstructive pulmonary disease, *DNR* do-not resuscitate decision, *IgG* immunoglobulin G, *Q1* 1^st^ quartile, *Q3* 3^rd^ quartile

At presentation in the emergency ward, median CRP was 82 mg/L and median ferritin was 1158 µg/L. One patient (5%) was diagnosed with an acute pulmonary embolism (PE) upon presentation in the emergency ward two weeks after start of COVID-19. In this case, low molecular weight heparin was started immediately. In echocardiography, cardiac function was uncompromised, with no evidence of hemodynamic consequences of PE. The patient was in good clinical condition when discharged to home with oxygen treatment, and there were no signs of ongoing, clinically significant inflammation. Baseline characteristics and other laboratory parameters upon hospital admission are further presented in Table 2.

### Ward treatment prior to remote treatment and monitoring

The median duration of hospitalization was six days. Three patients (16%) were also treated in intensive care unit. The duration of intensive care was 11, 12 and 15 days, respectively. The maximum level of provided respiratory support was invasive mechanical ventilation in two cases (11%) with an intubation period of seven days for both patients. Non-invasive ventilation with a bi-level positive airway pressure ventilator was applied as the maximum level of respiratory support in five patients (26%). With the remaining 12 patients (63%), low-flow nasal oxygen supplementation was the maximum level of respiratory support applied. All nineteen patients received oral glucocorticoid for hypoxemic COVID-19, seventeen patients (89%) received prophylactic low molecular weight heparin, and tocilizumab was administered in five patients (26%). One patient (5%) received nirmatrelvir/ritonavir as antiviral treatment. None of the patients had received anti-SARS-CoV-2 monoclonal antibody treatment. Data of treatment during hospital admission are presented in Table 3.

**Table 3:** Characteristics of hospital admission prior to home treatment and monitoring

	Median value or n/N	Q1-Q3 or %
Duration of hospital admission, days	6	4 – 18
Admitted in the Intensive Care Unit	3/19	16%
Highest C-reactive protein during admission	104	72 – 180
Highest level of Ferritin during admission	1410	603 – 1793
Drugs received for COVID-19		
Systemic corticosteroids	19/19	100%
Tocilizumab	5/19	26%
Nirmatrelvir/ritonavir	1/19	5%
Other antiviral medication	0/19	0%
Low-molecular weight heparin	17/19	89%
Maximum ventilation support received		
Low-flow nasal oxygen supplement	12/19	63%
Non-invasive ventilation	5/19	26%
Invasive mechanical ventilation	2/19	11%
Complications		
Clostridioides difficile infection	1/19	5%
Other bacterial infections		
Serratia marcescens lower respiratory tract infection	1/19	5%

### Home oxygen treatment and monitoring

**Fig. 2:**
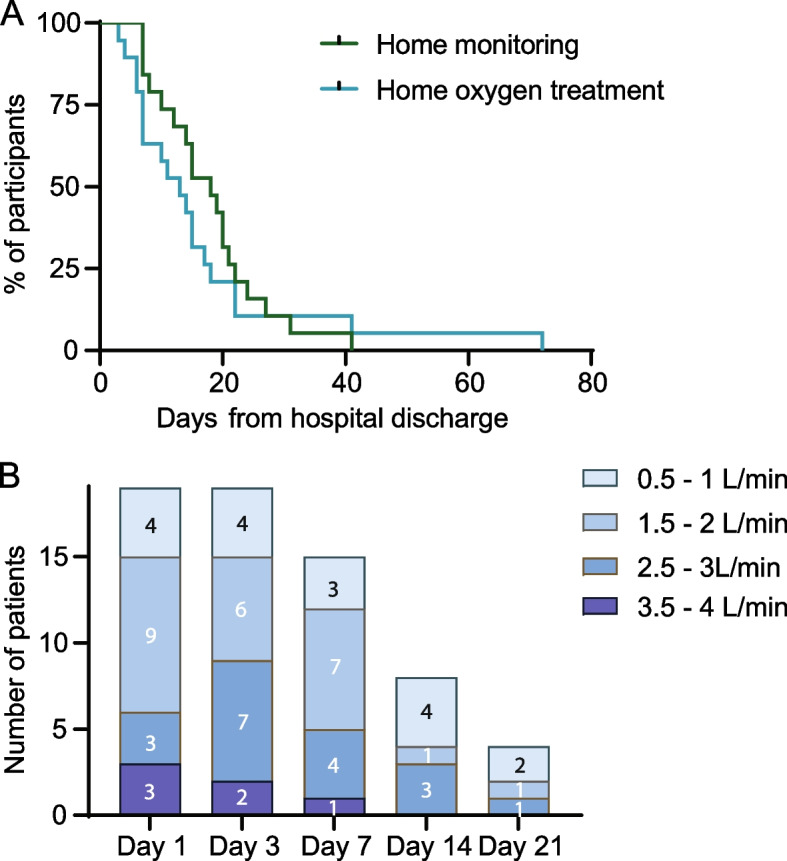
Remote oxygen treatment. **A** survival curve of the duration of remote oxygen treatment (blue) and monitoring (green) from hospital discharge. In two cases, home monitoring could be stopped as the patients were clinically stable although still in need of oxygen treatment. **B** numbers of patients with ongoing remote oxygen treatment grouped according to their oxygen flow levels

The median duration of monitoring was 18 days ranging from 7 to 41 days. The median duration for the oxygen treatment was 13 days with a range of 3 to 72 days. For one patient, monitoring was discontinued after 42 days despite ongoing oxygen therapy, as the patient was otherwise stable. The demographic of follow-up and oxygen treatment are presented as Kaplan-Meyer curves in Fig. 2A. All patients had need for oxygen supplementation upon discharge. The median oxygen flow at the beginning of home treatment was 2 L/min with a range of 1 to 4 L/min, as depicted in Fig. 2B. During the follow-up, median of daily lowest peripheral oxygen saturation-measurements was 89%. At the sixth day of monitoring, median CRP was 3 mg/L, median leukocyte count 9.2 × 10^9^/L and the median D-dimer 0.5 mg/L. Findings during the monitoring are displayed in Table 4.

**Table 4:** Ventilatory support and laboratory parameters during home oxygen treatment and monitoring program

	Median value or n/N	Q1-Q3 or %
Duration of oxygen treatment, days	19	13 – 18
Duration of monitoring, days	18	10 – 22
Oxygen flow at entry in the program, L/min	2	1 – 2
Oxygen flow at day 7, L/min	2	1 – 2.5
Last leukocytes count before entry in the program, × 10^9^/L	7.5	6.4 – 10.1
Leukocyte count at day 6, × 10^9^/L	9.2	6.7 – 15.1
Last C-reactive protein before entry in the program, mg/L	14	3 – 33
C-reactive protein at day 6, mg/L	5.0	2.0 – 20
Last ferritin before entry in the program, µg/L	984	495 – 1268
Ferritin at day 6, µg/L	802	362 – 1229
Last D-dimer before entry in the program, mg/L	0.5	0.1 – 1.7
D-dimer at day 6, mg/L	0.5	0.3 – 0.5

During the home monitoring, one patient developed pancreatitis due to increased use of alcohol after completing oxygen treatment. No other complications or safety issues were reported during monitoring.

### Cost–benefit analysis

The median number of avoided hospital treatment days was 9 days per patient with a range of 6 to 24 days. The total number of hospital treatment days prevented was 210.5 days. Of these, 9 would have been isolation days and 201.5 non-isolation days according to hospital protocol. Therefore, the total prevented costs of hospitalization were €179,465. Expenditure related to the home monitoring program added up to €34,975, mostly caused by the development of the application by a commercial partner (€34,570). Due to the relatively small number of recruited patients, monitoring could be performed within the routine working schedules without additional working force. As displayed in Table 5, the net benefit was €144,490 for 19 patients, i.e., €7,605 per patient on average.

**Table 5:** Cost–benefit assessment of the development and use of the home oxygen treatment and monitoring program

			Costs (-) and savings ( +)
Costs of application			
Development costs and usage costs	€ -34 570		€ -34 570
Costs of measurement devices	Per unit	Units (n)	
Pulse oximeters	€ 38.49	6	€ -231
Blood pressure monitors	€ 29	6	€ -174
Prevented hospitalization	Per day	Days (n)	
In isolation	€ 910	9	€ + 8 190
Not in isolation	€ 850	201.5	€ + 171 275
Total savings—costs			€ + 144 490

Costs are expressed as negative saldo and savings are presented as positive saldo. Due to confidentiallity, costs related to the development and the use of the application could not be further specified. Hospitalization days are the mean value of estimations by two pulmonologists, as described in the methods-section

## Discussion

In our case series of 19 COVID-19 patients, post-hospitalization home oxygen treatment and mobile-based monitoring facilitated early hospitalization of hypoxemic patients during the Omicron wave of the COVID-19 pandemic. This resulted in some relief to the overwhelmed healthcare as well as significant saving of healthcare expenditure.

Home monitoring of COVID-19 patients has been applied in different settings and in different ways; pre-hospitalization or post-hospitalization, with or without oxygen treatment, symptom-based or vital signs based [6–15].

Safety of a similar post-hospitalization monitoring program supporting home oxygen treatment was also established in a cohort of 73 COVID-19 patients from Paris, France, in the first three months of the pandemic when the wild type was the dominant variant of SARS-CoV-2 [9]. In a retrospective study of a virtual ward in 2021 in the United Kingdom, 3/44 patients discharged with an oxygen concentrator were readmitted and 1/44 died [10].

With high uncertainty how the Omicron wave would affect healthcare in Finland, we anticipated on the possibility of high numbers of prolonged hospitalizations. Indeed, the number of hospitalized patients has been high throughout the year 2022. A total of 1 124 195 confirmed or probable COVID-19 cases and a total of 8 238 new inpatient episodes due to COVID-19 in secondary care [3] were registered in Finland in 2022, while a much larger proportion remained undiagnosed after testing policy was adjusted due to restricted diagnostic capacity [3, 16].

The main objectives of the implementation of our program were to increase flexibility of the healthcare service, to reduce the number of hospitalized COVID-19 patients and to relieve workload at a critical phase of the pandemic. With 210.5 prevented hospitalization days, mostly around the early peak of the Omicron wave, this goal was achieved. Similarly, home oxygen treatment was associated with 6.4 ± 3.2 days reduction of hospital admission in patients who were discharged with oxygen therapy in comparison to the hospital protocol according to a cohort study of 320 COVID-19 patients from the Netherlands, until May 2021, likely including mainly wild-type and alpha variants cases [11]. In a study performed in California, United States, in 2021, a remote patient monitoring program was associated with a shorter length of hospital stay in the intervention group of 75 patients in comparison to a control group (median 4.8 versus 6.1 days; *p* = 0.03) [12]. However, in a randomized controlled trial, including 62 patients, only a small, non-significant difference of 1.6 hospital-free days was observed in favor of the intervention group [13]. Several factors may contribute to the difference between their findings and ours. In their intervention group, mean duration of home oxygen treatment was only 6.7 days after randomization, which suggests differences in severity of COVID-19 in patients selected for remote treatment, or in substantial differences in treatment regimes. Remote monitoring may also result in slower tapering of oxygen and possibly overestimation of prevented hospital days in our study. Furthermore, the investigators of that study found that in their study setting, early discharge disseminated to their control group, as these patients had also been informed about the intervention.

In our study, only few patients were enrolled in the later phase of the Omicron wave, as the patient population and the clinical picture of hospitalized COVID-19 patients changed to a more aged patient group with a phenotype without severe lung involvement. This was potentially due to age-dependent immunity dynamics upon vaccination and/or previous exposure to COVID-19 [3, 16]. This raises the question if there is any large-scale need for home oxygen treatment for future COVID-19 patients.

Even though our study population was rather small, the savings were significant due to prevented hospitalization days, on average 7,605 euro per patient. In Ireland, ambulatory monitoring protocol was applied in a cohort of 502 COVID-19 patients with a cost–benefit estimates ranging from net costs to health service of €142,000 to net savings of €27,883 depending on admission rate to home monitoring during the first months of the pandemic [14]. Similarly to our study, post-hospitalization home oxygen treatment was found to be both safe and economically beneficial in a Dutch study consisting of 49 COVID-19 patients [15]. In that study, the potential reduction in hospital days was 616 days in total or 12.6 days per patient and the estimated costs avoided were €146,736. Readmission rate was 12%.

Cost–benefit of post-hospitalization monitoring of COVID-19 patients may depend on several factors, such as the selection of patients, epidemiologic aspects, local and national healthcare costs as well as costs related to development and utilization of the monitoring programs. Furthermore, the costs analysis depended on projected discharge dates, based on clinical parameters as reported by the patient via the application. The estimation of reduction in hospitalization may be inaccurate, and hospitalization itself may also predispose to complications such as nosocomial infections [17]. Therefore, the cost–benefit evaluation should be interpreted with caution and cannot be generalized. However, even with uncertainty about the exact amount saved, our findings strongly indicate that post-hospitalization monitoring of hypoxemic COVID-19 is economically beneficial. Moreover, financial costs and financial benefits may be prone to multiple disturbing factors. The financing model may require modification in order to optimize the utilization of hospitalization-reducing telemedicine programs.

Apart from the benefits of home treatment, the program also has its limitations. For instance, the utility of application-based monitoring can be limited by poor technical or language skills and may thereby cause inequity in received healthcare. In some cases, the patients were provided with a tablet (iPad) to allow the involvement of family members or home care nurses in the use of the application while preserving the patients’ privacy. Furthermore, even though home treatment may relieve the burden of hospitalized patients, implementation of a new working tool and preparing and educating of patients for monitoring may require extra effort and time from the treating staff and increase the experienced working load.

Our study has some limitations. First, only a modest number of patients were enrolled in our program. Therefore, the risk of clinical deterioration and other safety issues may be underestimated. The small sample size may also lead to under- or overestimation of costs. Second, our data cannot be generalized due to several specific aspects mentioned in the previous paragraph. Costs of hospitalization may differ significantly between countries, which hampers comparison of hospitalization costs at the international level [18]. Third, as previously discussed, retrospective estimation of reduction of the length of hospital stay is rather insecure. Prospective, randomized studies are practically challenging in rapidly evolving epidemics with acute need of saving human resources and are still limited by the impossibility of blinding. Fourth, this study was not designed to evaluate the possible limitations of the program mentioned above. Nevertheless, we feel that our study contributes to the evidence that remote oxygen treatment and monitoring of COVID-19 patients can be safely performed and can be highly cost-effective.

Future studies and reports are needed to address whether remote home oxygen treatment and monitoring remains beneficial during the later phase of the epidemic and to evaluate whether these programs can safely be applied in other conditions, such as pneumonia of different etiologies, exacerbation of chronic pulmonary conditions, and pulmonary embolism. Furthermore, the prevalence of persisting symptoms of patients enrolled in such programs could be assessed, preferably in prospective studies, as a large proportion of hospitalized COVID-19 patients report persisting symptoms. Thus, we think that a randomized trial on rapid discharge supported by remote monitoring with or without oxygen treatment could be feasible in respiratory infections beyond COVID-19.

## Conclusions

From our study we conclude that home treatment and remote home monitoring can be safe and economically beneficial in carefully selected patients. This provides proof of concept that post-hospitalization monitoring can be successfully implemented in post-acute pulmonary conditions, possibly also beyond COVID-19. In epidemiologically less dynamic conditions, prospective studies to evaluate telemedicine programs may be more feasible.

## Data Availability

The datasets used and/or analysed during the current study are available from the corresponding author on reasonable request.

## Abbreviations

CRP: C-reactive protein
IQR: Interquartile range
SARS-CoV-2: Severe acute respiratory syndrome coronavirus 2
